# Bacterial profile of aggressive periodontitis in Morocco: a cross-sectional study

**DOI:** 10.1186/s12903-015-0006-x

**Published:** 2015-02-24

**Authors:** Hanane Chahboun, Maria Minguez Arnau, David Herrera, Mariano Sanz, Oum Keltoum Ennibi

**Affiliations:** EREB (Research Group of Oral Ecosystem), Faculty of Medicine Dentistry, Mohammed V University, Rabat, Morocco; ETEP (Etiology and Therapy of Periodontal Diseases) Research Group, Faculty of Dentistry, University Complutense, Madrid, Spain

**Keywords:** Periodontal pathogens, Aggressive periodontitis, Chronic periodontitis, Subgingival microbiota, *A. actinomycetemcomitans*

## Abstract

**Background:**

Aggressive periodontitis (AgP) is one of the most severe forms of periodontal diseases. In Morocco, *Aggregatibacter actinomycetemcomitans* has been strongly associated with AgP, however limited knowledge is available about the implication of other periodontal pathogens in this entity. Therefore, the main aim of this study was to evaluate the composition of the subgingival microbiota in Moroccan patients with AgP.

**Methods:**

Subgingival plaque samples were collected from 50 aggressive, 13 localized and 37 generalized periodontitis patients. Samples from 20 chronic periodontitis (ChP) patients were taken as controls. Samples collected from the four deepest periodontal pockets in each patient were pooled in pre-reduced transport fluid and examined by culture.

**Results:**

*A. actinomycetemcomitans* was significantly more frequent (p = 0.004) in generalised AgP compared to ChP, and *Porphyromonas gingivalis* was less prevalent in localized AgP, when compared with generalized AgP (p = 0.040) or ChP (p = 0.016). *Prevotella intermedia*, *Fusobacterium nucleatum* and *Tannerella forsythia* were also frequently detected in all groups. Mean proportions of *A. actinomycetemcomitans* were significantly higher in AgP groups, when compared to ChP, and generalized AgP patients harbored significantly higher proportions of *P. gingivalis* and *T. forsythia*, when compared to localized AgP or ChP.

**Conclusions:**

*A. actinomycetemcomitans*, *P. gingivalis*, *T. forsythia*, *P. intermedia* and *F. nucleatum* were frequently detected in this Moroccan population with AgP. Differences in frequency of detection, counts and proportions of *A. actinomycetemcomitans, P. gingivalis* and *T. forsythia* suggests the presence of distinct microbiological profiles for localized AgP, generalized AgP and ChP patients.

## Background

Aggressive periodontitis (AgP) is a form of periodontitis characterized by rapid and severe periodontal destruction in otherwise young healthy individuals. The etiology of periodontitis is very complex including the dental biofilm, which triggers the immuno-inflammatory response in a susceptible host. This interaction leads to the destruction of the periodontal tissues [1,2]. Pathogenic bacteria are the primary etiology agents in the pathogenesis of periodontitis. The oral biofilm is very complex and count for over 700 species, however only some micro-organisms have been specifically associated with periodontal diseases [3]. The majority of periodontal pathogens are Gram-negative and strict anaerobe, acting in synergy. Among the most important species, *Aggregatibacter actinomycetemcomitans* has been frequently associated with AgP [4,5]. Other bacteria are known as been associated with progression of periodontal destruction, such as *Porphyromonas gingivalis, Tannerella forsythia, Treponema denticola, Fusobacterium nucleatum, Prevotella intermedia*, *Prevotella nigrescens, Campylobacter rectus, Eikenella corrodens* and *Parvimonas micra* [6]. All these bacterial species produce a wide variety of virulent factors enabling them to colonize subgingival sites, to resist the defense mechanisms of the host and to cause periodontal tissue destruction [7].

These micro-organisms do not suffice to advance the disease. Indeed, the host immune response modules the evolution of the disease toward destruction or cure [8]. The role of these bacteria in the pathogenesis of the human periodontitis is based on their high frequency of isolation, the ability to adhere to epithelial cells, the ability to produce numerous virulent factors like the extracellular matrix proteins, protease, collagenase, endotoxin (LPS), bacteriocins, hemotactic inhibitors, leucotoxins, cytotoxins, toxic metabolic substances (H_2_S, putricines), immunosuppressive proteins, etc. [9,10].

Whereas *A. actinomycetemcomitans* is widely associated with localized AgP [7,11], *P. gingivalis* is regarded as the major causative agent in chronic periodontitis (ChP) [12]. Recently, a very strong association between the presence of the JP2 clone of *A. actinomycetemcomitans* and AgP was demonstrated among adolescents in Morocco [13]. In addition, a prospective longitudinal study has shown that infection with the JP2 clone is likely to be important in the initiation of the disease [14]. However, other periodontopathic bacteria, such as *P. gingivalis* are also suspected of participating in AgP [15]. It is admitted that AgP is not a mono-infectious disease, and the bacterial profile of this entity has not been studied in depth in Morocco. Thus, identification of the most prevalent bacteria is necessary to understand the bacterial etiology and could help to understand the treatment failure in some cases.

The aim of this study was to characterize the subgingival microbiota in a Moroccan population with AgP, including the evaluation of the presence and quantification, by means of culturing, of *A. actinomycetemcomitans*, *P. gingivalis*, *F. nucleatum, C. rectus, P. intermedia/nigrescens, T. forsythia, E. corrodens, P. micra, Eubacterium* spp., and *Capnocytophaga* spp.

## Methods

### Study population

This cross-sectional study was performed with a convenience sample of consecutive patients seeking treatment at the clinical department of Periodontology at the Faculty of Dentistry of Mohammed V Souissi University, Rabat, Morocco (from January 2011 to December 2012).

All subjects were verbally informed about the investigation, and they were asked to sign informed consent. The study was approved by the Medical Ethical Committee of Mohammed V Souissi University, Rabat, Morocco.

Patients, who met the inclusion criteria and sign the informed consent, were entered into the study. All patients had at least 20 teeth, diagnosed as having AgP or ChP, and were ≤ 35 years of age. The exclusion criteria were patients medically compromised, or having received periodontal or antibiotic treatment within the preceding 6 months, patients under orthodontic treatment, pregnant women or during lactation, and patients who need antibiotic prophylaxis before screening.

### Clinical and radiographic examination

The following clinical variables were scored at six sites per tooth, and in all teeth except third molars: dichotomous plaque presence, bleeding on probing (BOP), probing pocket depth (PPD, assessed to the nearest millimeter using a standard periodontal probe), clinical attachment level (CAL, calculated as the distance from the cemento-enamel junction to the bottom of the periodontal pocket).

A full mouth periapical radiographic examination was also performed to confirm by evidence interproximale bone loss. Bone loss was estimated by determining the ratio of the distance from the cemento-enamel junction to the alveolar bone crest. All clinical data was collected by the same examiner.

The diagnosis for the periodontal status was established for all subjects based on the 1999 International Classification of periodontal diseases and conditions [16]. The used clinical criteria were as follows. For aggressive periodontitis: 1) rapid attachment loss and bone breakdown were evident including at least one incisor and one first molar, 2) pocket depth ≥4 mm, clinical attachment loss ≥3 mm, presence of bleeding on probing. For chronic periodontitis: extensive deposits of plaque and calculus, more than 10% of teeth with pocket depth ≥4 mm, and at least one site having attachment loss ≥3 mm.

### Bacterial sampling and analysis

Sampling collection was done within week after interviewing and clinical examination, by means of pooled subgingival samples from each patient. Subgingival plaque samples were collected from four deep sites, one per quadrant, from each patient.

Supragingival plaque, located in direct proximity to sample sites, was carefully removed using a scaler and cotton gauze. A sterile, absorbent paper point was gently inserted into the apical extent of the periodontal pocket (sulcus). After 20 seconds, the papers were pooled in a tube containing 1.5 ml of reduced transport fluid (RTF) medium [17]. All plaque samples were collected by the same examiner.

### Microbiological procedures

Samples were transferred to the laboratory of Microbiology and Molecular Biology, Faculty of Dentistry, University of Complutense Madrid, Spain, within 24 hours, where they were homogenized by vortexing for 30 s [18], and serially diluted in phosphate-buffered saline (PBS). At the laboratory, aliquots of 0.1 ml were plated manually for the detection of *A. actinomycetemcomitans* on a specific medium [19]. These plates were incubated for 3 days in air with 5% CO_2_ at 37°C. Suspected isolates were identified on the basis of colony morphology (small colony, 1 mm in diameter, with a dark border and a “star” or “crossed cigars” shaped inner structure) and positive catalase reaction. Sample dilutions were also plated onto a non-selective blood agar plate (Blood Agar Base II®, Oxoid, Basingstoke, UK), supplemented with haemine (5 mg/l), menadione (1 mg/l) and 5% sterile horse blood. After 7–14 days of anaerobic incubation (80% N_2_, 10% CO_2_ and 10% H_2_), total counts and counts of representative colonies (those with colony morphologies compatible with target pathogen morphology) were performed in the most suitable plates, those harboring between 30 and 300 colonies. Suspected colonies were further identified by microscopy, studying gram staining and enzyme activity (including N-acetyl-β-D-glucosaminidase, α-glucosidase, α-galactosidase, α-fucosidase, esculin, indole and trypsin-like activity). In addition, the black pigmented colonies of *P. gingivalis* and *P. intermedia* have been tested under red fluorescent light UV (360 nm): negative for *P. gingivalis* and positive for *P. intermedia* [20]. Counts were transformed in colony-forming units per milliliter of the original sample. Total anaerobic counts were calculated, as well as counts of the detected periodontal pathogens (*A. actinomycetemcomitans, T. forsythia, P. gingivalis, P. intermedia/nigrescens, P. micra, C. rectus, E. corrodens. Eubacterium* spp.*, Capnocytophaga* spp., and *F. nucleatum*). In addition to the quantitative microbiological data, the frequency of detection and proportions for each bacterial species were also calculated.

### Data analysis

Different groups were compared: AgP and ChP, on one side; and localized and generalized AgP on the other side. The patient was used as experimental unit for observation. Demographic and clinical data were calculated for each subject (mean ± standard deviation, when needed the median was use). Differences in demographic and clinical parameters among groups were established with the one-way analysis of variance test. Chi square test and Fisher’s exact test were employed to compare the frequency of detection of different pathogens between groups. For counts of bacterial species, colony forming units were log transformed to achieve a normal distribution and ANOVA was used as a primary test to compare the three groups; as post hoc test, multiple rank tests were conducted when differences were detected. For proportions of anaerobic microflora, the Kruskal Wallis test was used primarily, while differences were explored post hoc by Mann–Whitney test. *P* values < 0.05 were considered statistically significant.

## Results

A total of 70 subjects were recruited in this study, divided in 50 cases of AgP (13 localized, 37 generalized) and 20 of ChP.

### Clinical findings

In the comparison between AgP and ChP, demographic and clinical data of patients are summarized in Table 1. The mean age of patients was significantly lower in AgP than in ChP group (p < 0.001). Pockets were significantly deeper in AgP than in ChP patients (p < 0.001), and CAL was significantly higher for AgP (p < 0.001).

**Table 1:** **Demographic and clinical data in aggressive (localized and generalized) and chronic periodontitis groups**

	**Localized aggressive periodontitis**	**Generalized aggressive periodontitis**	**Chronic periodontitis**	P*
	**(LAgP)**	**(GAgP)**	**(ChP)**	
	**n = 13**	**n = 37**	**n = 20**	
**Gender: female/male (% female)**	11/2 (84.6%)	29/8 (78.4%)	16/4 (80.0%)	>0.05
**Age: mean ± standard deviation (range)**	19.85 ± 4.616 (13; 26)	24.43 ± 5.058 (17; 36)	28.55 ± 4.347 (21; 35)	<0.001
**Plaque index: median (range)**	36.6% (25.9 - 72.5)	80.4% (53.9 - 100.0)	56.2% (45.1 - 76.0)	0.001
**Bleeding on probing: median (range)**	30.2% (23.1 - 68.9)	76.8% (59.1 – 100.0)	46.4% (33.7 - 59.9)	0.000
**Probing pocket depth: mean ± standard deviation**	6.23 ± 1.25	6.05 ± 0.95	4.46 ± 0.59	<0.001
**Clinical attachment level: mean ± standard deviation**	6.03 ± 1.94	5.18 ± 1.39	3.09 ± 1.20	<0.001

*P value for multiple comparisons by means on ANOVA. Statistically significant differences corresponded to.

Age: LAgP vs GAgP, p = 0.006; LAgP vs ChP, p <0.001; GAgP vs ChP, p = 0.003.

Plaque index: LAgP vs GAgP, p = 0.001; GAgP vs ChP, p = 0.008.

Bleeding on probing: LAgP vs GAgP, p < 0.001; GAgP vs ChP, p <0.001.

Probing pocket depth: GAgP vs ChP, p < 0.001; LAgP vs ChP, p < 0.001.

Clinical attachment level: GAgP vs ChP, p < 0.001; LAgP vs ChP, p < 0.001.

In the comparison between localized and generalized AgP, significantly more sites with plaque and gingival inflammation were observed in generalized AgP (p < 0.05) (Table 1).

### Microbiological findings

#### Frequency of detection

In the comparison between AgP and ChP, a tendency towards statistically significant differences was detected regarding the presence of *A. actinomycetemcomitans*: 60.0% in AgP versus 25.0% in ChP (p = 0.08). In both groups, *P. gingivalis*, *P. intermedia*, *F. nucleatum* and *T. forsythia* were frequently detected, with no statistically significant differences; however, *P. gingivalis* was more frequent in AgP than in ChP (82,0% versus 60,0%, respectively). *Capnocytophaga* spp., *P. micra*, and *C. rectus* showed lower frequencies in both groups. *A. actinomycetemcomitans* was significantly more frequent in generalized AgP than in ChP (p = 0.004). Also *P. gingivalis* was more prevalent in GAgP when compared with both LAgP (p = 0.040) and ChP (p = 0.016) Table 2.

**Table 2:** **Frequencies of detection [n positive (percentage)] of studied bacteria in aggressive periodontitis compared to chronic periodontitis**

	**Aggressive periodontitis**			**Chronic periodontitis**
	**Localized aggressive periodontitis**	**Generalized aggressive periodontitis**	**All aggressive periodontitis**	
	**n = 13**	**n = 37**	**n = 50**	**n = 20**
A.actinomycetemcomitans*	6 (46.2%)	24 (64.9%)	30 (60.0%)	5 (25.0%)
P. gingivalis*	8 (61.5%)	33 (89.2%)	41 (82.0%)	12 (60.0%)
***P. intermedia/nigrescens***	11 (84.6%)	32 (86.5%)	43 (86.0%)	18 (90.0%)
***T. forsythia***	6 (46.2%)	24 (64.9%)	30 (60.0%)	11 (45.0%)
***P. micra***	1 (7.7%)	11 (29.7%)	12(24.0%)	4 (20.0%)
***C. rectus***	2 (15.4%)	5 (13.5%)	7(15.0%)	3 (15.0%)
***F. nucleatum***	10 (76.9%)	31(82.4%)	41(82.0%)	16 (80.0%)
***Capnocytophaga*** **spp.**	4 (30.8%)	10 (27.0%)	14(28.0%)	7 (35.0%)
***E. corrodens***	6 (46.2%)	9 (24.3%)	15(30.0%)	3 (15.0%)

*Statistically significant differences were detected, by means of Chi-square or Fisher Exact test, for.

*A.actinomycetemcomitans*: AgP vs ChP, p = 0.080; GAgP vs ChP, p = 0.004.

*P. gingivalis:* GAgP vs ChP, p = 0.016; GAgP vs LAgP, p = 0.040.

#### Proportions of anaerobic microflora

Differences in mean proportions of *A. actinomycetemcomitans* were statistically significant among groups (p = 0.004), corresponding to lower proportions in ChP when compared to localized AgP (tendency, p = 0.062) and generalized AgP (p = 0.001). For *P. gingivalis*, means proportions were also significantly different among groups (p = 0.038), when higher values for generalized AgP when compared to localized AgP (tendency, p = 0.064) and ChP (p = 0.014). Finally, significant differences among groups for *T. forsythia* (p = 0.028) corresponded to significant higher counts in generalized AgP, as compared to localized AgP (p = 0.027) and ChP (p = 0.045) Table 3.

**Table 3:** **Proportions (expressed as mean and standard deviation -sd) of total anaerobic microflora, isolated by culture in localized, generalized aggressive and chronic periodontitis**

	**Localized aggressive periodontitis**		**Generalized aggressive periodontitis**		**Chronic periodontitis**		K-W*
	**(LAgP) n = 13**		**(GAgP) n = 37**		**(ChP) n = 20**		
	**mean**	**sd**	**mean**	**sd**	**mean**	**sd**	**p value**
***A. actinomycetemcomitans***	18.16%	29.10%	11.55%	38.81%	0.05%	0.18%	0.004
***P. gingivalis***	12.49%	15.21%	28.25%	27.04%	13.78%	18.39%	0.038
***P. intermedia/nigrescens***	5.78%	7.07%	4.92%	7.20%	4.78%	7.97%	0.598
***T. forsythia***	1.32%	2.58%	5.52%	8.32%	3.03%	7.76%	0.028
***P. micra***	0.09%	0.34%	0.91%	2.40%	1.18%	2.96%	0.298
***C. rectus***	0.55%	1.79%	0.08%	0.26%	0.10%	0.34%	0.958
***F. nucleatum***	1.96%	3.52%	1.43%	2.11%	1.90%	1.59%	0.266
***Capnocytophaga*** **spp.**	0.67%	1.59%	0.93%	3.08%	0.12%	0.25%	0.902
***E. corrodens***	0.44%	0.87%	0.28%	1.00%	0.18%	0.40%	0.457

*Kruskal-Wallis test was used to compare the three groups; detected differences were explored by means of Mann Whitney test to compare two groups. Differences corresponded to.

*A.actinomycetemcomitans*: LAgP vs ChP, p = 0.062; GAgP vs ChP, p = 0.001.

*P. gingivalis*: LAgP vs GAgP, p = 0.064; GAgP vs ChP, p = 0.014.

*T. forsythia*: LAgP vs GAgP, p = 0.027; GAgP vs ChP, p = 0.045.

#### Total anaerobic counts and counts of specific pathogens

Total anaerobic counts were significantly different among groups (p = 0.011), and the differences corresponded to higher levels in generalized AgP when compared to the other two groups. Also, significant differences among groups were detected for *A. actinomycetemcomitans* (p = 0.019) and *P. gingivalis* (p = 0.030), associated to lower counts in ChP as compared to the AgP groups, and to higher counts for generalized AgP as compared to ChP, respectively Table 4.

**Table 4:** **Total anaerobic counts and counts of selected bacterial species (in logarithm, expressed as mean and standard deviation –sd) in localized, generalized and chronic periodontitis**

	**Localized aggressive periodontitis**		**Generalized aggressive periodontitis**		**Chronic periodontitis**		ANOVA*
	**(LAgP) n = 13**		**(GAgP) n = 37**		**(ChP) n = 20**		
	**mean**	**sd**	**mean**	**sd**	**mean**	**sd**	**P value**
***Total counts***	8.41	0.69	8.90	0.62	8.47	0.52	0.011
***A. actinomycetemcomitans***	2.62	2.99	3.32	2.62	0.81	1.47	0.019
***P. gingivalis***	3.93	2.81	5.37	2.35	3.52	3.01	0.030
***P. intermedia/nigrescens***	4.35	2.14	4.79	1.85	3.95	1.99	0.297
***T. forsythia***	2.11	2.79	3.81	2.88	2.44	2.61	0.084
***P. micra***	0.39	1.42	1.47	2.33	1.03	2.13	0.290
***C. rectus***	0.79	1.94	0.61	1.58	0.69	1.69	0.939
***F. nucleatum***	3.15	2.23	4.05	1.88	3.98	1.82	0.337
***Capnocytophaga spp.***	1.28	2.03	1.41	2.22	1.23	1.95	0.953
***E. corrodens***	1.60	2.16	1.09	2.19	0.94	1.71	0.655

*ANOVA test was used to compare all groups, and multiple range tests to identify the explanation of the detected differences.

Total counts: GAgP, versus LAgP and ChP.

*A. actinomycetemcomitans*: ChP, versus LApP and GAgP.

*P. gingivalis* : GAgP versus ChP.

## Discussion

The results of the present cross-sectional study has shown distinct microbiological profiles for different types of periodontitis, namely localized and generalized AgP and ChP:. *A. actinomycetemcomitans* was significantly more frequent in generalized AgP than in ChP, and *P. gingivalis* was also more prevalent in generalized AgP than in both localized AgP and ChP. Other significant differences among conditions were detected for proportions of anaerobic microflora for *A. actinomycetemcomitans*, *P. gingivalis* and *T. forsythia*, and for total anaerobic counts and counts of *A. actinomycetemcomitans* and *P. gingivalis*.

Conversely, for other pathogens, similar results were found, such as for *P. intermedia*, *Capnocytophaga* spp., *F. nucleatum*, *P. micra* or *C. rectus*. These results may support previous data showing that, when considered as a whole, the subgingival microbiota may not differ significantly between AgP and ChP [21-26]. And, as showed in earlier worldwide studies on periodontal microbiota, this study confirm the presence and interspecies relationships of periodontopatic bacteria [11,27-29]. Ximenez-Fyvie et al. [26] employed the checkerboard DNA-DNA hybridization technique to describe the subgingival microbial composition of 77 Mexican subjects and found that the microbial differences between generalized AgP and generalized ChP subjects were only discrete and none of the 40 bacterial species tested seemed to specifically differentiate the subgingival microbial profile of either periodontitis groups. Takeuchi et al. [25] used polymerase chain reaction (PCR) to determine the prevalence and culture to study the proportion of seven subgingival species in samples from 93 Japanese subjects with AgP, ChP or healthy conditions. A significantly higher percentage of generalized AgP and generalized ChP were carriers of *C .rectus*, *P. gingivalis*, *T. forsythia* and *Treponema denticola* than periodontally healthy subjects. And the proportions of *A. actinomycetemcomitans*, *P. gingivalis* and *T. forsythia* were similar in all periodontitis groups. Heller et al. [30] evaluated the presence of 40 bacteria by the means of checkerboard DNA-DNA hybridization technique, on Brazilian population (75 individuals with AgP and 185 with ChP). They found that only few species differed between AgP and ChP, *P. gingivalis* and *T. denticola* were related to ChP, while *Eubacterium nodatum* was associated with AgP. The same authors reported that *A. actinomycetemcomitans* was very frequent in ChP and AgP and significant differences of *A. actinomycetemcomitans* between the two forms of disease are related to specific clone type or serotypes and not only the presence or levels of this pathogen. Mombelli et al. [23] evaluated whether the presence or absence of *A. actinomycetemcomitans*, *P.gingivalis*, *P.intermedia*, *T.forsythia*, and *C. rectus* could distinguish between ChP and GAgP. They concluded that these bacteria presented a limited interest in discriminatory ability to identify subjects with GAgP or ChP. However, a diagnosis of GAgP was more likely in subjects positive for Aa than subjects negative for this bacterieum. Moreover, the highly leukotoxic strain was uniquely associated with AgP. Mahalakshmi et al. [31] reported to that in addition to *A. actinomycetemcomitans*; the prevalence of *P.gingivalis* and *T. forsythia* was also high in periodontitis and while *A. actinomycetemcomitans* showed a positive association with AgP while T.denticola was associated with ChP.

In the present study, statistically significant differences regarding the frequency of detection were reported: e.g. *A. actinomycetemcomitans* was more frequently isolated in AgP than in ChP (60.0% versus 25.0%; p = 0.080), supporting previous data showing that this bacterial species is highly associated with AgP in Morocco, either when using culture or PCR as a detection method [32,33]. In addition, despite the importance of *A. actinomycetemcomitans* in periodontitis, in the present report it was a frequent finding the co-occurrence of two or more species of the studied bacteria within the same patient. Ashimoto et al. [34] reported that there is a symbiotic relationship between micro-organisms in periodontal pockets. Indeed, a pathogen may colonize subgingival sites already occupied by other bacteria. Or some bacteria may occur together in periodontal lesions because they both produce destructive disease without interacting with each other.

*P. gingivalis* is highly prevalent among subjects with ChP [35-38]. In AgP, its frequency varies between 62% and 100% in different populations [25,39,40]. In the present study, *P. gingivalis* was isolated in 82.0% and 60.0% of AgP and ChP, respectively, and the differences were statistically significant. These results suggest that *P. gingivalis* may be also an important pathogen in AgP patients in Morocco. The presented frequencies are similar to the frequencies reported in Colombia (74.6% and 68.2%, respectively) [39] or Chile (88.8% and 76.4%, respectively) [41].

In the present research, the frequency of *T. forsythia* isolation was slightly higher in AgP compared to ChP. Botero et al. [42] reported a frequency of 50% in both forms of periodontitis in Colombia. Many studies suggested that *T. forsythia* is associated with progressive periodontitis [43-45] and “refractory” periodontitis [46,47]. More recently, Tomita et al. [48] studied the prevalence and levels of Aa, Pg and T. forsythia in subgingival plaque samples of a group of 40 Japanese patients with aggressive and chronic periodontitis (20 AgP and 20 ChP). The authors reported that no distinct pattern of the subgingival bacteria profile was discerned between the two entities, except for T. forsythia which was significantly higher in ChP subjects than in AgP subjects. They added also that bacterial account for Pg and T. forsythia were positively correlated with pockets depth and clinical attachment loss which corroborate the role of those bacteria belonging to the red complex in pathogenesis of periodontitis.

Based on the clinical examination (PPD, CAL) and radiographic patterns of periodontal destruction, AgP patients were subdivided into localized or generalized. The most frequent pathogens in both conditions were *P. gingivalis* (61.5%), *P. intermedia* (84.6%) and *F. nucleatum* (76.9%), for localized AgP, and. *P. gingivalis* (89.2%), *P. intermedia* (86.5%), *T. forsythia* (64.9%), and *F. nucleatum* (82.4%), for generalized AgP. Lee et al. [22] used PCR to determine the prevalence of seven putative periodontal pathogens in 39 Korean AgP patients and reported that all the studied bacteria, *A. actinomycetemcomitans* (75%), *P. gingivalis* (96.8%), *P. intermedia* (78.8%), *T.* forsythia (94.2%), *Fusobacterium* spp. (99.4%), *Treponema* spp. (96.8%) and *Parvimonas micra* (85.9%), were frequently associated with AgP in this population. In particular *P. intermedia* was significantly associated with generalized AgP. In Japan, subgingival samples were collected from 50 patients with AgP (10 localized, 40 generalized) and 35 samples from generalized ChP. *T. forsythia* and *P. gingivalis* were detected more frequently at sites where the attachment loss was severe (CAL ≥ 6 mm), in comparison with those with moderate attachment loss (CAL < 6 mm) in patients with localized AgP. This trend was also observed for *P. gingivalis*, among patients with generalized AgP. However, the positive correlation between the presence of *C. rectus/T. denticola* and the severity of the clinical attachment loss was not found in any of the three groups of periodontitis [25]. Kowalski et al. [49] reported that high frequency detection of *A. actinomycetemcomitans* and *C. rectus* in generalized Ag periodontitis. In our study *C. rectus* showed low occurrence either in aggressive periodontitis and chronic periodontitis it titers were low to in the two entities.

In our study, when studying the counts and proportions of cultivated bacteria, *P. gingivalis* and *T. forsythia* showed a stronger association to generalized AgP in comparison with either localized AgP or ChP. Thus, these results may suggest that *P. gingivalis* could play a major role in the pathogenesis of generalized AgP in this population. Conversely, no significant differences in the frequency of detection, counts or proportions were detected in the present study, between localized and generalized AgP for *A. actinomycetemcomitans.* These results are in contrast with previous data in a Moroccan population, using PCR, which showed that the presence of *A. actinomycetemcomitans* was significantly associated with localized Ag P compared to generalized AgP [32].

The previously mentioned discrepancy could be explained in part by the fact that PCR is more sensitive for bacterial detection, with lower detection thresholds (and localized AgP may harbor lower amounts of the target bacteria, as shown in Table 4). But also, by the fact that in the present study, the sampling strategy included a pooled subgingival sample of the four deepest pockets (one per quadrant) and, therefore, the occurrence of pathogens may have been underestimated. Other explanation could be the small sample size for localized comparing to generalized AgP. It should be also highlighted that culture cannot distinguish between high and low virulent strains of *A. actinomycetemcomitans*. Culture investigations have shown that the level of subgingival cultivated microbiota *A. actinomycetemcomitans* is lower than those of *P. gingivalis* in adult patients with periodontitis [50]. The difference between the levels of *A. actinomycetemcomitans* and *P. gingivalis* was also demonstrated by Rams et al. [29] who examined subgingival samples of 1,800 patients with periodontitis. The average proportion of *A. actinomycetemcomitans* in the microbiota was 4%, while that of *P. gingivalis* was 23%. Compared to other periodontal bacteria, such as *P. intermedia/nigrescens, C. rectus* or *P. micra*, the proportion of *P. gingivalis* was also higher [29].

Depending on criteria that a bacterium of total sub gingival cultivated flora above a certain minimal threshold to be associated with periodontitis, it has been suggested that this threshold is 0.1% for *P. gingivalis* [51], 0.01% for *A. actinomycetemcomitans* [36,51], 2.5% for *P. intermedia* [43,51], 5% for *F. nucleatum* and *Capnocytophaga* spp*.* [43,52], 1% for *E. corrodens* [43,52], and 2% for *C. rectus* [43,53].

Differences detected in the present study may not represent true differences in the population. The differences in percentages of detection of periodontal pathogens found worldwide between studies may be due to geographical differences, socio-economic status as well as to the genetic factor of the host. It is to note also that a strict comparison is difficult because of the detection method and laboratory standards may differ between team researchers, clinical criteria used in studies may be different to, mild or severe, localized or generalized periodontal breakdown. However, as mentioned by Lourenco et al. [54], a microbial consortium combining *A.actinomycetemcomitans* and other potential pathogens may be helpful to discriminate between AgP and CP. Further studies on large samples are needed. Microbiological aspect of periodontitis and especially aggressive periodontitis is peace of the complex multifactorial etiology that should be taken in consideration to help in better mentoring and treatment of this entity.

## Conclusions

This study confirms the association of *A. actinomycetemcomitans* with localized and generalized AgP in Morocco, while *P. gingivalis* was highly associated with generalized AgP. Differences in frequency of detection, counts and proportions of *A. actinomycetemcomitans, P. gingivalis* and *T. forsythia* suggests the presence of distinct microbiological profiles for localized AgP, generalized AgP and ChP patients. More studies are necessary to understand the role of these bacterial species in aggressive periodontitis pathogenesis.
